# A study on HIV, Syphilis, and Hepatitis B and C virus infections among female sex workers in the Republic of Congo

**DOI:** 10.1186/s13690-017-0189-5

**Published:** 2017-05-08

**Authors:** Fabien Roch Niama, Nadia Claricelle Loukabou Bongolo, Pembe Issamou Mayengue, Franck Fortuné Mboussou, Edith Sophie Kombo Bayonne, Florian Michael Kouckodila Nzingoula, Louis Regis Dossou-Yovo, Igor Louzolo, Mandingha Kosso Etoka-Beka, Achile Lanzy, Irène Yameogo, Davy Louvouezo, Simon Charles Kobawila, Marie-Francke Puruhence, Henri Joseph Parra

**Affiliations:** 1 Laboratoire National de Santé Publique (LNSP), Unité de Biologie Moléculaire, avenue du Général De Gaulle BP: 120, Brazzaville, République du Congo; 2 Faculté des Sciences de la Santé, Université Marien Ngouabi, Brazzaville, République du Congo; 3 Secretariat Exécutif Permanent (SEP) du Conseil National de Lutte Contre le Sida: 2.459, Brazzaville, Congo; 4 Médecins d’Afrique, NGO, PO Box: 45, Brazzaville, Congo

**Keywords:** Female Sex Workers, HIV, HBV, HCV, Republic of Congo

## Abstract

**Background:**

Female Sex Workers (FSWs) are considered to be at high risk for transmission of Sexually Transmitted Infections (STIs) and are defined as a priority of the national HIV/AIDS response in the Republic of Congo (RoC). However, no data are available regarding STIs in this group. This study aimed to determine the prevalences of HIV, syphilis and hepatitis B and C among FSWs in five cities in the country.

**Methods:**

A cross-sectional study was conducted from November 2^nd^ 2011 to May 15^th^ 2012. Participants were recruited in Brazzaville, Pointe-Noire, Dolisie, Nkayi and Pokola using a respondent-driven sampling method.

**Results:**

A total of 805 FSWs were recruited with an average age of 28.31 ± 9.15 years. The overall prevalences of HIV, syphilis, HBV and HCV were 7.50%, 2.20%, 4.20% and 0.70%, respectively. The age groups 35–39 (20.51% [0%–36.93%], *p* = 0.0057) and greater than 40 years (16.67% [0%–34.93%], *P* = 0.016) were positively associated with behaviors at high risk of HIV infection. For syphilis, the most infected age group was the one greater than 40 years, at 6.25% ([1.06% –72.37%] *p* = 0.04). Pointe-Noire was the most infected city for syphilis and HBV, with 5.15% (*p* = 0.0061) and 4.22% (p˂0.001), respectively. No risk factors were associated with HCV infection. FSWs practicing in mobile prostitution sites had a significantly higher infection rate (2.1% [0%–11.09%] *p* = 0.04).

**Conclusion:**

This study shows that the prevalence of HIV and other STIs in FSWs is high. Therefore, a combination of individual and structural interventions could reduce the risk of an STI “reservoir” among this population.

## Background

HIV is responsible for one of the most destructive epidemics in recorded history. In 2015, UNAIDS reported 70% of the people living with HIV worldwide, 70% of new HIV infections and 66% of people dying from AIDS. Sub-Saharan Africa remains the most heavily affected region, and in this region, women represent more than 60% of HIV-infected persons [1]. Female sex workers (FSWs) are considered to be a high-risk group for the acquisition of HIV and other sexually transmitted infections (STIs). The relationship between sex work and HIV vulnerability has been recognized since the earliest days of the epidemic [2]. In countries with a generalized epidemic like the Republic of Congo (RoC), the HIV prevalence is consistently higher among FSWs than in the general population, and it was estimated that FSWs are 13.5 times more likely to be infected than other women [1]. To our knowledge, in Central Africa the burden of syphilis and hepatitis is not well described, with the exception of certain groups such as blood donors. Indeed, in the RoC approximately 7.5% and 0.5% of donors are affected by hepatitis B and C, respectively [3].

A systematic review of behavioral risk factors for STIs in FSWs in sub-Saharan Africa shows that the vulnerability of FSWs is inextricably linked to the realities of this “profession”. Accordingly, STI interventions for FSWs in the RoC are not guided by knowledge of country-specific risk factors, as recommended by UNAIDS in their practical guidelines for intensifying HIV prevention towards universal access [4]. The vulnerability for HIV as well as other STIs, such as syphilis and hepatitis viruses, in the FSW group is primarily the consequence of a high number of sexual partners and a high frequency of unprotected sex. Indeed, several previous studies have shown an association between STI prevalences among FSWs and inconsistent condom use as the main risk factor for HIV infection [4–6]. It has also been found that FSWs have a higher risk of contracting STIs from their non-paying partners than from their clients [2, 7].

In the RoC, FSWs have been identified as one of the highest-risk populations and priorities of national AIDS responses [8]. Unfortunately, there has been no study conducted in this group determining STI prevalences. Therefore, the current study aimed to determine prevalences of HIV, syphilis, and hepatitis B and C infections and to identify associated risk factors for these infections among FSWs in the RoC.

## Methods

A cross sectional study was conducted from November 2^nd^ 2011 to May 15^th^ 2012in fixed and mobile FSW sites in five cities of the RoC, including Brazzaville, Pointe-Noire, Dolisie, Nkayi, and Pokola.

### Data collection

Participants were recruited using the respondent-driven sampling technique, which has been shown to be reliable for conducting studies in hard to reach groups and samples [9]. Mapping was done to identify FSW sites, followed by the enumeration of FSWs at each site by trained interviewers in bars and nightclubs, with the help of neighborhood leaders, police officials, youth, and the associations of FSW against HIV/AIDS. The total number of FSWs and the number of sites per type of prostitution are shown in Table 1. The average number of FSWs per site was estimated to be 11.

**Table 1:** Total number of different type of prostitution and FSW per sites

Cities	Total nomber of stes			Total Nomber of FSWs		
	Fixed Sites	Mobiles sites	Total	Fixed Sites	Mobile sites	Total
Brazzaville	58	59	117	670	483	1 153
Pointe-Noire	12	134	146	180	1 383	1 563
Dolisie	13	9	22	54	39	93
Pokola	6	3	9	180	145	325
Nkayi	0	3	3	-	79	79
Total	89	208	297	1084	2129	3 213

In the absence of baseline data on HIV prevalence in this population, the required sample size was calculated using the following formula and by taking as reference the mean of the ratio “HIV prevalence in this population/HIV prevalence among those aged 15–49 in Central Africa,” which was estimated at 18.7%:

n = [z_α_^2^*p*(1-p)/e^2^]*d. Where z_α_ = reduced risk α = 5%; e: margin of error which is set at 0.05, p: estimated prevalence of HIV infection among FSWs; d: FSWs Inclusion Interval.

The prevalence of HIV among people aged 15 to 49 years is 3.20%. By applying a sampling interval of 3 and by taking into account a predicted refusal rate of 15%, the sample size was estimated at 845 as described in Table 2. The total number of sites necessary to achieve the estimated sample size was calculated by taking into account the average number of FSWs by site according to the mapping results as described in Table 1.

**Table 2:** Distribution of sex worker prostitution sites by cities

Cyties	Total	Fixed Sites	Mobiles sites	Number of FSWs
Brazzaville	25	12	13	301
Pointe-Noire	30	3	27	276
Dolisie	4	2	2	92
Pokola	2	1	1	88
Nkayi	1	0	1	88
Total	62	18	44	845

The total number of sites required to reach the estimated sample size was calculated by taking into account the average number of FSW per site according to the mapping results, which was 11. Thus, the total number of sites needed to reach the required sample size was estimated at 62. Table 3 shows the distribution of sites by city and by category according to the distribution of these variables in the population as a whole.

**Table 3:** Number of selected sites per cities

Cities	Number of seleted sites		
	Fixed sites	mobile sites	Total
Brazzaville	12	13	25
Pointe-Noire	3	27	30
Dolisie	2	2	4
Pokola	1	1	2
Nkayi	0	1	1
Total	18	44	62

FSWs attending each site were invited, in groups of 20, to attend a workshop dedicated to STI education and data collection for the study. At the second visit, the workshop was organized as beneficial for the FSWs. Each workshop started with a presentation of the objectives, activities and expected results, followed by asking for willingness to participate in the study and an invitation for individual face-to-face interviews. Questionnaires were administered using an individual questionnaire adapted from one developed by Family Heath International for behavioral surveys [10]. FSWs who agreed to participate in the study were then asked to provide consent.

FSWs were defined as adults or young females who received goods in exchange for sexual services, either regularly or occasionally, and who may or may not consciously define those activities as income-generating [11].

### Laboratory procedures

Five milliliters (5 mL) of whole blood were collected in EDTA tubes. Plasma was then obtained after centrifuging for 10 min at 5000 rev/min and kept at −20 °C before transfer to the Molecular Biology Unit of Laboratoire National de Santé Publique (LNSP) in Brazzaville, where all the HIV, HCV, HBV and syphilis tests were performed. Plasma specimens were screened for HIV and HCV antibodies and the surface hepatitis B antigen (AgHBs) using ELISA tests (Bio-Rad Genscreen HIV1/2 Ag-Ac Ultra, Bio-Rad Monolisa HCV and Bio-Rad Monolisa HBV Ultra, Marne la Coquette, France, respectively). The following algorithm was used to detect antibodies against HIV: the ELISA-positive samples were confirmed by the Western blot (New LAV Blot I, Bio-Rad, Marnes la Coquette, France). The Rapid Plasma Reagine (RPR) test (BioScan, BHA BIO-TECH India) was first used to detect syphilis infection, and active syphilis was identified by testing the RPR-positive samples with the TPHA test (Biotech TPHA test kit, UK).

### Data analysis

All data were entered into Cs Pro version 4.0 and then transferred to SPSS 17.0 software for statistical analysis. Differences with a *p*-value of <0.05 were considered statistically significant. The analyzed variables included the prevalences of syphilis, HIV, Hepatitis B and C. The prevalence’s of these infections were tested for associations with cities (Brazzaville, Pointe-Noire, Dolisie, Nkayi and Pokola), age group (<20, 20–24, 25–29, 30–34, 35–39 and >40 years old), age of first sex (<15 and > 15 years old), drug use and unprotected sex. Differences between proportional data were compared using the chi-square test, a multivariable logistic regression model was used and the magnitude of associations was assessed using odds ratios with respective 95% CIs.

## Results

Out of a total of 852 FSWs interviewed, 805 (94.4%) were enrolled, including 35.6% (*n* = 287) in Brazzaville, 28.9% (*n* = 233) in Pointe-Noire, 11.5% (*n* = 93) in Dolisie, 11.6% (*n* = 94) in Nkayi and 12.17% (*n* = 98) in Pokola. The percentages of FSWs originally from the RoC, the Democratic Republic of Congo (DRC) and other countries were, respectively, 59.2%, 38.8%, and 2%.

### Sociodemographic information, history in sex work, knowledge of STIs and sexual behavior characteristics

The average age of the participants was 28.3 ± 9.1 years old (ranging from 18 to 66 years). FSWs spent an average of 19.7 ± 4.5 years in sex work. Among the FSWs interviewed, 8.6% were unschooled, 24.6% had primary education and 66.9% had secondary education. Approximately 18.7% of FSWs were sharing life with a partner versus 81.3% who were living alone. The percentage of FSWs using home-based sex work was 31.6%, while 58.2% were using establishment-based sex work and 2.2% mixed the two types of sex work.

### Prevalences of HIV, Syphilis, HBV and HCV

The highest prevalences of HIV were observed in the cities of Pointe-Noire (12.8%) and Dolisie (7.5%), which also had prevalences of active syphilis of 5.1% and 2.1%, respectively. Concerning HBV and HCV, the highest prevalences were found in Ponte-Noire (7.3% and 14.1%, respectively), followed by Brazzaville for HCV (7.3%) and Nkayi for HBV (6.3%). In all 5 cities together, the prevalences of HIV, syphilis, HBV and HCV were 7.5%, 2.2%, 4.2% and 0.7% respectively, as described in Table 4.

**Table 4:** prevalence of HIV, syphilis (passive and active) and hepatitis B and C depending on cities

		Cities									
		Brazzaville		Dolisie		Nkayi		Pointe Noire		Pokola	
		n	% [IC 95 ]	n	% [IC 95 ]	n	% [IC 95 ]	n	% [IC 95 ]	n	% [IC 95 ]
HIV	Negative	270	94,1 [91,26 – 96,8]	86	92,5 [86,9 – 98]	91	96,8 [93,2 – 100]	206	88,4 [84,04 – 92,7]	95	96,9 [93,47 – 100]
	Positive	17	5,9 [0,00 – 17,1]	7	7,5 [0,00 – 27]	3	3,2 [0,00 – 23 ]	30	12,8 [0–23,6]	3	3 [0,00 – 22,5]
RPR	Negative	278	96,9 [94,82 – 98,9]	89	95,7 [91,48 – 99,9]	88	93,6 [88,51 – 98,7]	214	91,8 [88,18 – 95,5]	86	87,7 [80,83 – 94,6]
	Positive	9	3,1 [0,00 – 14,5]	4	4,3 [0,00 – 24,1]	6	6,3 [0,00 – 25,9]	19	8,1 [0,00 – 20,4]	12	12,2 [0,00 – 30,7]
TPHA	Negative	285	99,3 [98,34 – 100]	91	97,8 [94,87 – 100]	93	98,9 [96,85 – 100]	221	94,8 [91,94 – 97,7]	97	98,9 [96,98 – 100]
	Positive	2	0,7 [0,00 – 12,2]	2	2,1 [0,00 – 22,2]	1	1 [0,00 – 21,1]	12	5,1 [0,00 – 17,6]	1	1,0 [0,00 – 20,7]
HCV	Negative	286	92,7 [89,55 – 95,8]	92	95,7 [91,48 – 99,9]	94	95,7 [91,57 – 99,9]	229	85,8 [81–90,6]	98	97,9 [95,13 – 100]
	Positive	1	7,3 [0,00 – 18,4]	1	4,3 [0,00 – 24,1]	0	4,2 [0,00 – 24]	4	14,1 [2,27 – 26]	0	2 [0,00 – 21,6]
AgHBs	Negative	282	98,2 [96,73 – 99,7]	90	96,7 [93,12 – 100]	88	93,6 [88,51 – 98,7]	216	92,7 [89,24 – 96,1]	95	96,9 [93,47 – 100]
	Positive	5	1,7 [0,00 – 13,2]	3	3,2 [0,00 – 23,2]	6	6,3 [0,00 – 25,9]	17	7,3 [0,00 – 19,6]	3	3 [0,00 – 22,5]

### Factors associated with HIV, Syphilis, HBV and HCV status

Risk factors were investigated and multivariable analyses showed that the risk of contracting HIV and syphilis infections increased with age, specifically with age greater than 34 and 40 years (COR = 4.72 CI: 1.57 –14.15; p <0.005 and COR = 8.76 CI: 1.06 to 72.3; *p* = 0.044, respectively). Additionally, prevalences of syphilis and hepatitis B were significantly higher for FSWs living in the city of Pointe-Noire (COR = 14.19 [2.13-94.45]; *p* = 0.006 and COR = 6.61 [2.21–19.75; *p* = 0.0007 respectively]), and practicing mobile prostitution was associated with a greater risk of syphilitic infection (COR = 0.1 [0.01–0.91]; *p* = 0.014).

Other factors, including drug use, age at first sexual intercourse, the systematic use of condoms or type of prostitution, were not associated with the occurrence of these infections (Table 5).

**Table 5:** VIH, syphilis, hepatitis B and C infections associated with sociodemographic factors

HIV					Syphilis			HBV			HCV		
Variables	N	Reactive n (% [CI])	cOR (CI 95%)	P value	Reactive n (% [CI])	cOR (CI 95%)	P value	Reactive n (% [CI])	cOR (CI 95%)	P value	Reactive n (% [CI])	cOR (CI 95%)	P value
Cyties													
Brazzaville	287	17 (5,92% [0% – 17%])			2 (0,7% [0% – 12,2%])			5(1,7% [0% – 13,2%])			1 (0,35% [0% – 11,9%] )		
Dolisie	93	7 (7,5% [0% – 27%])	1,3 [0,5 – 3,7]	0,53	2 (2,1% [0% – 22,2%])	6,9 [0,8 – 62]	0,08	3 (3,2% [0% – 23,2%])	2,1 [0,46 – 9,3]	0,33	1 (1,08% [0% – 21,3%] )	6,88 [0,1 – 257,7]	0,29
Nkayi	94	3(3,19% [0%–23% ])	0,6 [0,16 – 2,2]	0,46	1 (1,06% [0% – 21,1%])	0,84 [0,05 – 13]	0,90	6 (6,3% [0% – 25,9%]	3,4 [0,93 – 12,4]	0,06	0 (0% [0% – 0%] )	0	-
Pointe Noire	233	30(12,8% [0%–23,6%])	1,8 [0,89 – 3,7]	0,09	12 (5,1% [0% – 17,6%])	14,2 [2,13 – 94,4]	0,006	17 (7,3% [0% – 19,6%])	6,6 [2,21 – 19,7]	0,0007	4 (1,7% [0% – 14,4%])	28,22 [0,5 – 1365,9]	0,09
Pokola	98	3 (3,06% [0% – 22,55%] )	0,5 [0,14 – 2]	0,36	1 (1,02% [0% – 20,7%])	2 [0,1–1]	0,59	3 (3% [0% – 22,5%])	1,9 [0,42 – 9,1]	0,38	0 (0% [0% – 0%] )	0	-
Group age (years)													
< 20	142	6 (4,23% [0% – 20,32%] )			2 (1,41% [0% – 17,7%] )			9 (6,3% [0%–22,2%])			0		
20 –24	200	7 (3,5% [0% – 17,11%] )	1 [0,31 – 3,3]	0,978	4 (2% [0% – 15,72%] )	3,7 [0,44 – 31,4]	0,22	8 (4% [0% – 17,5%])	0,8 [0,29 – 2,3]	0,72	1 (0,5% [0% – 14,3%])	0	-
25 –29	180	5 (2,78% [0% – 17,18%] )	0,7 [0,19 – 2,5]	0,58	1 (0,56% [0% – 15,1%] )	0,8 [0,05 – 12,4]	0,87	9 (5% [0% – 19,2%])	0,9 [0,32 – 2,5]	0,84	1 (0,5% [0% – 15,1%] )	0	-
30 –34	109	10 (9,17% [0% – 27,07%] )	2,4 [0,8 – 7,6]	0,11	3 (2,75% [0% – 21,2%] )	4,2 [0,46 – 38]	0,20	4 (3,6% [0% – 22,1%])	0,62 [0,18 – 2,2]	0,46	0	0	-
35 et 39	78	16 (20,51% [0% – 36,93%] )	4,7 [1,57 – 14,1]	0,00	2 (2,56% [0% – 24,4%] )	2,8 [0,26 – 31,2]	0,39	1 (1,2% [0% – 23,3%])	0,18 [0,02 – 1,5]	0,11	1 (1,2% [0% – 23,3%] )	0	-
> 40	96	16 (16,67% [0% – 34,93%] )	3,8 [1,28 – 11,3]	0,01	6 (6,25% [0% – 25,6%])	8,7 [1,06 – 72,3]	0,04	3 (3,1% [0% – 22,8%])	0,44 [0,1 – 1,8]	0,26	3 (3,1% [0% – 22,8%] )	0	-
Age of first sexe													
< 15 years	309	20 (6,47% [0% – 16,95%] )			10 (3,2% [0% – 14,2%])			16 (5,1% [0% – 16%])			3 (0,97% [0% – 12,07%] )		
> 15 years	496	40 (8,06% [0% – 16,12%] )	1 [0,58 – 2]	0,80	8 (1,6% [0% – 10,3%] )	0,4 [0,14 – 1,1]	0,07	18 (3,6% [0% – 12,2%])	0,75 [0,36 – 1,5]	0,43	3 (0,6% [0% – 9,3%] )	0,39 [0,06 – 2,42]	0,31
Drug use													
No	683	51 (7,46% [0% – 14,26%] )			14 (2,05% [0% – 9,4%] )			27 (3,9% [0% – 11,3%])			4 (0,59% [0% – 8,06%] )		
yes	122	9 (7,38% [0% – 24,45%] )	1,1 [0,49 – 2,77]	0,73	4 (3,28% [0% – 20,7%] )	1,4 [0,34 – 6,2]	0,61	7 (5,7% [0% – 22,9%])	1,5 [0,55 – 4,3]	0,40	2 (1,64% [0% – 19,2%] )	6,7 [0,83 – 53,82]	0,07
Unprotected sex													
No	364	36 (9,89% [0,14% – 19,64%] )			10 (2,7% [0% – 12,8%] )			14 (3,8% [0% – 13,9%])			3 (0,82% [0% – 11%] )		
Yes	441	24 (4,76% [0% – 13,87%] )	0,4 [0,06 – 4,02]	0,49	8 (1,8% [0% – 11,0%] )	0	-	20 (4,5% [0% – 13,6%])	0,87 [0,1 – 7,8]	0,89	3 (0,68% [0% – 9,9%] )	0	-
Condom use													
Never	46	4 (8,68% [0%–30,76%])			0 (0% [0% – 0%] )			1 (2,1% [0% – 30,7%])			0		
unconsistent	395	20 (5,06% [0% – 14,67%])	1,4 [0,18 – 12,25]	0,70	8 (2,03% [0% – 11,7%] )	0	-	19 (4,8% [0% – 14,4%])	1,9 [0,24 – 15,9]	0,53	3 (0,76% [0% – 10,6%] )	0	-
Always	364	36 (9,89% [0,14% – 19,64%])	0	-	10 (2,7% [0% – 12,8%] )	0	-	14 (3,8% [0% – 13,9%])	0		3 (0,82% [0% – 11,05%] )	0	-
Injecting drug use													
No	778	55 (7,07% [0,3% – 13,84%])			16 (2,06% [0%–9,01%] )			31 (3,9% [0% – 10,8%])			6 (0,7% [0% – 7,7%] )		
Yes	27	5 (18,51% [0%–43,7%])	0,5 [0,07 – 3,49]	0,49	2 (7,41% [0% – 43,7%] )	0,7 [0,06 – 9,71]	0,83	3 (11,1% [0% – 46,6%])	0,9 [0,14 – 6,9]	0,99	0 (0% [0% – 0%] )	0	-
Type of prostitution													
Home-based sex-work	255	22 (8,63% [0% – 20,36%] )			6 (2,35% [0% – 14,4%] )			11 (4,3% [0% – 16,3%])			3 (1,1% [0% – 13,3%] )		
Establishment-based sex-work	469	26 (5,54% [0% – 14,34%] )	0,9 [0,41 – 2,39]	0,97	10 (2,1% [0% – 11%] )	0,1 [0,01 – 0,91]	0,04	20 (4,2% [0% – 13,1%])	0,3 [0,09 – 1,3]	0,12	2 (0,4% [0% – 9,4%] )	0,03 [0–1,3]	0,06
Mixed	63	8 (12,69% [0% – 31,63%] )	1,3 [0,44 – 4,13]	0,59	-	0	-	0 (0% [0% – 0%] )	0		1 (1,6% [0% – 26,0%] )	2,84 [0,2 – 40,7]	0,44
Dont now	18	4 (22,22% [0% – 62,96%] )	10 [1,91 – 52,73]	-	2 (11,1% [0% – 54,6%] )	5,2 [0,36 – 76,93]	-	3 (16,6% [0% – 58,8%])	1,9 [0,25 – 15,8]		-	-	-

## Discussion

This first report on the prevalences of HIV, syphilis, HBV and HCV among FSWs in the RoC shows that the prevalence of HIV among FSWs is 7.5%, significantly higher than those among other women and in the general population [8]. Regarding the cities of Brazzaville, Dolisie and Pointe-Noire, prevalences of 4.9%, 7.5%, and 12.8% were reported, and these are above the national average (3.2%). This prevalence is lower than those reported in studies conducted in sub-Saharan countries. Indeed, in West Africa, prevalences of 33.7% and 22.9% were found in Cote d’Ivoire and Senegal, respectively [12, 13], whereas in Rwanda (East Africa) the HIV prevalence among FSWs was 59.8% [14]. In central Africa, where few studies are available, it was reported that 23.6% and 23.3% of FSWs were HIV-infected in Cameroon and the DRC (Democratic Republic of the Congo), respectively [15]. In the context of the RoC, these results suggest that, even in the context of a generalized epidemic, HIV infection remains hyper-endemic among FSWs and spreads to the general population through clients (customers) and regular partners [2], showing a role played by FSW partners as a potential bridge to the general population [16].

In this study, ages ranging beyond 34 years were positively associated with HIV infection in this population. This observation was reported in others studies assessing prevalence rates in Africa and abroad. Indeed, most of the HIV-reactive FSWs were reportedly in the 30–39-year-old age group in studies conducted in India, Burkina-Faso and Nigeria [17–19].

The current study has reported a relatively low prevalence of HCV among FSWs (0.74%). Literature data suggests a wide disparity of results in this population. In fact, while less than 1% among FSWs in Italy, the rate increases up to 74% among FSWs who inject drugs in China [20, 21]. Although in this study the use of drugs was almost nonexistent, it is unclear whether the low prevalence found in the present study reflects the real epidemic of HCV in this population in the RoC. Several studies conducted among non-injection drug users have established that the prevalence of HCV infection in this population was comparable to that observed in the general population. This observation has lead to the conclusion that sexual transmission is not necessarily a preferred route of transmission for HCV infection [21–23].

In this study, the prevalence of HBV was found to be 4.24%, lower than the prevalence reported in blood donors, where 7.5% of samples were hepatitis B surface antigen positive [3]. This low prevalence in this population in the RoC may be partly explained by the relatively low level of injection drug use, which was shown to be a risk factor for contamination among FSWs [24]. Additionally, the practice of anal sex by FSWs has also been shown to increase the probability of HBV infection [25]. In the current study, anal sexual intercourse was not documented. Therefore, the hypothesis that HBV is sexually transmitted in FSWs, including the correlation of exposure periods, should be investigated in the context of the RoC.

Little is known regarding syphilis infections in Africa in the subpopulation of FSWs. The current study reports a prevalence of 2.23% for this infection. This prevalence varies from one locality to another. Indeed, in Sudan the prevalence ranges from 1.5% to 8.9%, depending on the region of the country, and it was observed that the HIV and syphilis prevalences were similar [26]. The reason for this variability, especially of the larger vulnerability of FSWs living in the city of Pointe-Noire compared with other localities, remains unexplained. However, Pointe-Noire is a port city with significant economic activity, and has a significant epidemiological situation, as suggested by the data from FSWs in this study as well as data reported for other subgroups of the population (2009 Esic inquiry, investigation of HIV in pregnant women, 2012). The reasons for this feature are still unknown.

In this study, FSWs living in the city of Pointe-Noire were highly infected with HIV and hepatitis B virus in comparison with those living in Brazzaville. The reason for this difference is unknown.

### Limitations of the study

Certain limitations were noted in this study. The cross-sectional study design based on the respondent-driven sampling method, which is not necessarily representative of the FSW population, may be the origin of certain deficiencies, in particular as related to data concerning risk behaviors obtained on the basis of a questionnaire as this information is inherently difficult to verify. The existence of certain eligibility criteria may reduce the generalization of these data to all FSWs in the RoC. Additionally, the use of serological tests can introduce the possibility of false test results. For example, these tests cannot detect very recent infections, thus reducing their sensitivity, despite their sensitivity and specificity generally above 90% [27, 28].

## Conclusion

Despite HIV prevention programs being implemented that focus on them, FSWs remain a very high risk group and an important contributor to population-level HIV transmission in the RoC. This study was the first one assessing HIV, syphilis, HBV and HCV prevalences among FSWs. There is therefore an urgent need to put in place a second-generation STD surveillance system that can help document incremental outcomes and the impact of interventions for this specific population.

## Abbreviations

FSW: Female Sex Workers
HBV: Hepatitis B virus
HCV: Hepatitis C virus
HIV: Human Immunodeficiency Virus.
RoC: Republic of Congo
